# DSM-5 conduct disorder and symptoms in youths at high risk of psychosis in Kenya with DSM-5 mental disorders and substance use: towards integrated management

**DOI:** 10.1038/s41598-023-50192-3

**Published:** 2023-12-21

**Authors:** David M. Ndetei, Victoria Mutiso, Christine Musyimi, Reinpeter Momanyi, Pascalyne Nyamai, Peter Tyrer, Daniel Mamah

**Affiliations:** 1 Department of Psychiatry, University of Nairobi, Nairobi, Kenya; 2 Africa Mental Health Research and Training Foundation, Mawensi Road, Off Elgon Road, Mawensi Garden, P.O. Box 48423-00100, Nairobi, Kenya; 3 World Psychiatric Association Collaborating Centre for Research and Training, Nairobi, Kenya; 4 Imperial College, London, UK; 5 Department of Psychiatry, Washington University Medical School, St. Louis, MO USA

**Keywords:** Human behaviour, Risk factors

## Abstract

Little is known about the prevalence of Conduct Disorder (CD) and symptoms of CD in high risk psychosis persons at both clinical and community populations in LMICs and in particular Kenya. This study aimed to document (1) the prevalence of CD diagnosis and symptoms in youth who screened positive for psychosis and (2) the associated mental disorders and substance use in the same cohort in LMIC. The sample size was 536 students who had screened positive on the Washington Early Recognition Center Affectivity and Psychosis (WERCAP) from a population of 9,742 high school, college and university students, but had not converted to a psychotic disorder. We collected data on socio-demographic characteristics and used the following tools: Economic indicators tool; the Diagnostic Interview Schedule (DIS) tool for DSM-5 diagnosis; World Health Organization (WHO) Alcohol, Smoking, and Substance Involvement Screening Test (ASSIST). Basic descriptive statistics, chi-square test, Fisher's exact test, Pearson correlation and Poisson regression were conducted. Five percent (5%) of the respondents met the criteria for DSM-5 CD. Indeterminate CD comprised 10.1%. Male gender, all substances except hallucinogens lifetime, obsessive compulsive disorder, psychosis, agoraphobia, social phobia, drug abuse/dependence, antisocial personality disorder, oppositional defiant disorder, suicidality, WERCAP screen for bipolar disorder and WERCAP screen for schizophrenia were significantly (*p* < 0.05) associated with CD. Deceitfulness or theft criteria symptoms showed that CD had no significant gender difference. Criteria symptoms in aggression to people and animals, destruction of property and serious violations of rules were more common among males. Our findings suggest the need to screen for and diagnose CD, mental disorders and substance use in high risk psychosis youths in Kenya. This will inform integrated management.

## Introduction

Conduct disorder (CD), a relatively common condition in youth, has been well studied in many countries since the pioneering work of Lee Robins^1^. The relationships and co-morbidity of CD with psychotic conditions, depression and substance abuse, especially in people attending for treatment have been studied for a long time, especially in Western settings. In a USA based study on 26 bipolar youths aged 8–13, it was concluded that CD may co-exist with bipolar disorder (BD) at 69% lifetime comorbidity and 54% for episode comorbidity^2^. Bipolar 1 disorder and CD co-existence may represent a distinct subtype of either disorder in terms of presentation, course and treatments^3,4^. The combination of CD and BD in youth predicts high risks of substance use disorders in relatives suggesting a genetic predisposition^5^. Comorbidity of adolescent depression and/or anxiety and CD has also been demonstrated^6–9^ and so is comorbidity of depression, CD and drug abuse from adolescence to young adulthood^10^. There is also an increased prevalence of somatic problems in CD^11^. Understanding the role of CD in schizophrenia is of clinical significance, for example, factors contributing to schizophrenia that is preceded by CD include failing to learn not-to-behave aggressively in early childhood, impairments in understanding emotions in the faces of others, maltreatment, and subsequent re-victimization^12^. CD prior to age 15 is associated with an increase in aggressive behavior and crime among men with schizophrenia^13,14^. . A previous study indicated that in childhood and adolescence, CD was associated with poor performance, substance abuse and physical abuse, as well as an earlier age of onset of schizophrenia^15^. CD has also been found to be associated with antisocial personality disorder and substance abuse in schizophrenia^16^. CD predisposes to substance use initiation and/or co-occurrence with substance disorder^17–20^.

A recent systematic review of papers published between 1990 and 2010 on CD in 5–19 year olds found the prevalence figures of CD of 3.6% in males and 1.5% in females^21^. A more up to date report^22^ based on data obtained from the National Epidemiology of Iranian children and adolescents aged 6–18 (N = 30,532) found that (i) CD increased with age with prevalence of 0.58% in 6–9, 0.57% in 10–14 and 1.22% in 15–18 years, (ii) CD was more common in boys than in girls, (iii) CD was more likely if fathers had a psychiatric disorder, and (iv) up to 83.4% of CD cases met the criteria for at least one other psychiatric disorder; 54.89% had oppositional disorder; 32.34% had ADHD; 20.43% had tobacco use disorder and 18.3% had depressive disorders^22^. In general, most studies have confirmed the same gender differences; an early onset and high comorbidity with mental health disorders across the globe^23–26^. CD is also found in clinical populations with prevalence figures of up to 50% in all attendances of children and adolescents at health facilities^27,28^.

There is also evidence that the earlier the onset the worse the prognosis^29^ with 50–80% of the boys retaining the disorder for up to 4 years in follow up studies^30,31^ and with a higher risk of developing anti-social personality disorder^1,31,32^, aggressive behavior^33^, and higher comorbidity with different psychiatric disorders^5,34–37^ including substance use^24,38,39^. All of these comorbidities increase the cost of management^26,40–42^.

In Africa, a study in Nigeria found a high prevalence of CD of 14.5% in secondary school children aged 11–19^43^, much higher than the 1.79% found in the Iranian sample of 10–18 year olds^22^. In a Kenyan study of high school students from 2 different schools in their 1st, 2nd and 3rd years of study, the prevalence of CD was 34.7% (69/199) and 29.9% (123/412)^26^. In both schools, CD was more common in males than in females. This high prevalence and disparity in prevalence rates in the 2 schools could be a reflection of the communities and the catchment areas for those schools.

Several studies in Kenya have suggested a high prevalence of psychotic symptoms in youth populations in the community, underlining the need to do mass screening for psychosis in the general non-clinical populations^44–46^. In agreement with the global literature, we were not able to find any studies (in the last 5 years) that investigated CD in non-clinical populations with psychosis symptoms not yet converted i.e., high risk for schizophrenia. Understanding the comorbidity with other diagnoses is key to integrated management. In this study, we investigated CD, CD symptoms, mental disorders and substance use in a cohort of youth with psychotic symptoms but not yet converted. Such data are necessary in order for Kenyan mental health workers specializing in child and adolescent mental health to take a comprehensive and inclusive approach to any co-morbidity of schizophrenia and CD, including any co-existing substance use disorders. This combined management has the potential to reduce the chance of conversion from high risk to clinical schizophrenia and possibly improve the outcomes of management for each of the co-morbid conditions. Our study in Kenya will fill the gap on CD in high risk schizophrenia in Kenyan youth. Equally important, such data will contribute to the global database and inform researchers of any cross-cultural similarities and differences that could offer clues to prevention and management.

### The general objective and aims

The two main objectives of this study were to (i) determine the prevalence of DSM-5 conduct disorder (CD) in a cohort of Kenyan youth who had scored positive on the Washington Early Recognition Center Affectivity and Psychosis (WERCAP), (ii) determine the association of CD with mental disorders and substance use. The more specific aims were to determine in high risk schizophrenia:-

1. Prevalence of CD symptoms and diagnoses
2. Social demographics of the CD
3. Gender patterns in the CD symptoms
4. Comorbidity of mental disorders and CD
5. Comorbidity of substance use and CD

## Method

### Participants and procedure

This study was an immediate offshoot of a main study that screened 9,742 Kenyan youth from both rural and urban settings to determine how many of them were at high risk of schizophrenia but not yet converted to clinical schizophrenia. For this study, we used several tools:

1. *The Washington Early Recognition Center Affectivity and Psychosis (WERCAP).* This screen was originally developed to identify early symptoms of psychotic and bipolar disorders^47^. It is divided into two sections to estimate affectivity and psychosis severity based on symptom frequency and functionality. Developed in the United States, it has been found to have good psychometric properties in U.S youth with established mental disorders^48^. The WERCAP Screen has found applicability in LMICs specifically in Kenya^45^ and Rwanda^47,48^ in both clinical and non-clinical subjects with high test–retest reliability and validity with affectivity’s sensitivity of 0.91, specificity of 0.71 and psychosis sensitivity of 0.88 and specificity of 0.82. Nearly all the reported studies have been in youth with the aim of capturing the prodromal state of these conditions at the earliest possible time.
2. *A socio-demographic tool* to record age, gender, the highest level of education, marital status and birth order.
3. *The economic indicators tool* was used to record household items that estimate economic status by creating a wealth index^49^. The wealth index used is based on the World Bank recommendation for Low and Middle-Income Countries (LMICs)^49^ and has been adopted by the Kenyan Government for use in Kenya. It is classified into five sections; quintiles 1–5 with quintile 1 representing the lowest level of wealth and 5 the highest level. We have previously described the individual items of these economic indicators, their scores and the compilation of the wealth index^50^.
4. *For substance use/disorder we used the World Health Organization (WHO) Alcohol, Smoking, and Substance Involvement Screening Test (ASSIST)*: ASSIST has been developed by WHO and tested in several Low and Middle-Income Countries (LMIC) and recommended by WHO as a tool that collects information on and determines the presence or absence of different types of substance use and also levels of risk from the use of tobacco products, alcohol, cannabis, amphetamine-type stimulants, cocaine, sedatives and sleeping pills, hallucinogens, opioids, and “other” drugs^51^. It is a more comprehensive tool that enquires about the different types of substance use and uses a time pattern (current and lifetime) unlike DSM-5, which is more focused on abuse and dependence at the time of the interview. In this study, the tool was used only to determine the prevalence of different types of substance use on a ‘Yes’ or ‘No’ dimension. The WHO ASSIST has been used in Kenya^52^ and other countries in Africa^53^. It takes approximately 15 min.
5. *The Diagnostic Interview Schedule (DIS) for DSM-5 diagnosis*: The DIS^54^ is an online tool that has questions that are read to research participants by a trained research assistant who scores the responses and an algorithm that generates DSM-5 diagnosis^55^. The tool has a skipping mechanism and takes an average of 10 min. The research assistants/interviewers do not have to be clinicians to administer the tool. DIS has been found to have a high inter-rater reliability for diagnosing both the presence and absence of psychiatric disorders, including common externalizing and internalizing disorders^56^. This tool was administered to those students who scored positive for high-risk schizophrenia not yet converted to clinical schizophrenia. These students were drawn from the 9,742 screens of whom, 536 (5.5%; 536/9,742) scored positive for early psychosis. These 536 students consisted of 61 high school students (14.4%; 61/536), 96 college students (17.9%; 96/536) and 378 (70.7%, 378/536) university students.

### Preparation for the study and data collection

Training for data collection was done by one of us (DM from Washington University, St. Louis) on a face to face in person residential training in Kenya assisted by four researchers (a psychiatrist, public health physician, clinical psychologist and graduate nurse), who underwent training in person on the administration of the DIS at the University of Florida, USA conducted by the developers of the DIS. These acted as trainers of trainees of the research assistants in Kenya. The research assistants were trained in groups in a face to face in person training sessions.

Data collection was overseen by DMN & VM from the Africa Mental Health Research and Training Foundation (AMHRTF). The lead research assistant was a university Master Level graduate nurse who was responsible for the supervision of all sites and all data collection and transmission to AMHRTF headquarters. He supervised informed consent and/or consent/assent and sat through all data collection sessions. After obtaining institutional permission, the trained RA approached individual students in a private room to ensure confidentiality. The trained RA read each of the questions up to 3 times without elaboration of the questions and recorded the responses. All instructions were administered at the same time. Only those who scored positive on WERCAP were then given DIS and ASSIST.

### Data management and analysis

Data analysis was performed with SPSS version 21 (IBM, Chicago, IL). Basic descriptive statistics in the form of frequency, percentage, mean and standard deviation were done. Chi-square test or Fisher’s exact test when appropriate were used to analyze the difference in the proportion of conduct disorder symptoms with gender variable and the difference in prevalence between conduct disorder across different categories of socio-demographics, mental disorders and substance use variables. Differences in levels of continuous variables were examined using the Kruskal–Wallis test for non-parametric data. Significant socio-demographic, mental disorder and substance use variables were then fitted into the Generalized Linear Model (GLM) with Poisson distribution and log link function to identify the determinants of conduct disorder in the participants. The Poisson distribution accommodates well for count data and incorporates the assumption that the variance is equal to the mean. Statistical significance was considered at a *p*-value < 0.05.

### Ethics approval

Permission was sought from the local community administration for high school participants (their language of learning is English), and from the institutional heads for the tertiary academic institutions. The participants who were over 18 years old provided written informed and signed voluntary consent forms. For those under 18 years, written consent was obtained from the parents/guardians and assent from the students. Ethical approval was granted by the Maseno University Ethics Review Board in Kenya (IRB number MSU/DRPI/MUERC/00344/16).

### Ethics approval and consent to participate

Ethical approval was granted by the Maseno University Ethics Review Board in Kenya (IRB number MSU/DRPI/MUERC/00344/16). The authors assert that all procedures contributing to this work comply with the ethical standards of the relevant national and institutional committees on human experimentation and with the Helsinki Declaration of 1975, as revised in 2008*.* Participants who were over 18 years old provided written informed and signed voluntary consent forms. For those under 18 years, written consent was obtained from the parents/guardians and assent from the students.

## Results

All of the results are on DSM-5 CD and symptoms; DSM-5 psychiatric diagnosis and ASSIST substance use in students who had scored positive on WERCAP.

### Conduct disorder symptoms

Table 1 summarizes the prevalence of conduct disorder and its various symptoms. Overall, 5% of the respondents met the criteria for DSM-5 CD disorder. Indeterminate CD comprised 10.1% while negative CD comprised 84.9%.

**Table 1:** Prevalence of Conduct disorder symptoms in WERCAP positive students.

Symptoms of CD	Category	Total (N = 536)	Conduct disorder*		
			Negative	Indeterminate	All criteria met except exclusion for antisocial personality disorder
1. Often bullies, threatens, or intimidates others	No	482 (89.9%)	442 (97.1%)	28 (51.9%)	12 (44.4%)
	Yes	54 (10.1%)	13 (2.9%)	26 (48.1%)	15 (55.6%)
2. Often initiates physical fights	No	460 (85.8%)	428 (94.1%)	27 (50.0%)	5 (18.5%)
	Yes	76 (14.2%)	27 (5.9%)	27 (50.0%)	22 (81.5%)
3. Has used a weapon that can cause serious harm	No	499 (93.1%)	447 (98.2%)	39 (72.2%)	13 (48.1%)
	Yes	37 (6.9%)	8 (1.8%)	15 (27.8%)	14 (51.9%)
4. Has been physically cruel to people	No	495 (92.4%)	448 (98.5%)	30 (55.6%)	17 (63.0%)
	Yes	41 (7.6%)	7 (1.5%)	24 (44.4%)	10 (37.0%)
5. Has been physically cruel to animals	No	485 (90.5%)	434 (95.4%)	36 (66.7%)	15 (55.6%)
	Yes	51 (9.5%)	21 (4.6%)	18 (33.3%)	12 (44.4%)
6. Robbery (Stealing with confrontation)	No	531 (99.1%)	455 (100.0%)	50 (92.6%)	26 (96.3%)
	Yes	5 (0.9%)	0 (0%)	4 (7.4%)	1 (3.7%)
7. Has forced someone into sexual activity	No	530 (98.9%)	455 (100.0%)	49 (90.7%)	26 (96.3%)
	Yes	6 (1.1%)	0 (0%)	5 (9.3%)	1 (3.7%)
8. Has deliberately engaged in fire setting	No	530 (98.9%)	455 (100.0%)	52 (96.3%)	23 (85.2%)
	Yes	6 (1.1%)	0 (0%)	2 (3.7%)	4 (14.8%)
9. Has deliberately destroyed others' property	No	511 (95.3%)	450 (98.9%)	42 (77.8%)	19 (70.4%)
	Yes	25 (4.7%)	5 (1.1%)	12 (22.2%)	8 (29.6%)
10. Has broken into someone else's house or car	No	531 (99.1%)	454 (99.8%)	51 (94.4%)	26 (96.3%)
	Yes	4 (0.8%)	1 (0.2%)	2 (3.7%)	1 (3.7%)
	Indeterminate	1 (0.2%)	0 (0%)	1 (1.9%)	0 (0%)
11. Often lies to obtain favors or avoid obligations	No	408 (76.1%)	389 (85.5%)	14 (25.9%)	5 (18.5%)
	Yes	128 (23.9%)	66 (14.5%)	40 (74.1%)	22 (81.5%)
12. Thefts without confrontation: Shoplifting, fraud or Credit card or check forgery	No	510 (95.1%)	448 (98.5%)	40 (74.1%)	22 (81.5%)
	Yes	26 (4.9%)	7 (1.5%)	14 (25.9%)	5 (18.5%)
13. Often stays out late at night without permission before age 13	No	503 (93.8%)	444 (97.6%)	39 (72.2%)	20 (74.1%)
	Yes	33 (6.2%)	11 (2.4%)	15 (27.8%)	7 (25.9%)
14. Has run away overnight twice, or once without returning	No	504 (94.0%)	442 (97.1%)	45 (83.3%)	17 (63.0%)
	Yes	32 (6.0%)	13 (2.9%)	9 (16.7%)	10 (37.0%)
15. Often truant from school, beginning before age 13	No	449 (83.8%)	413 (90.8%)	28 (51.9%)	8 (29.6%)
	Yes	87 (16.2%)	42 (9.2%)	26 (48.1%)	19 (70.4%)
DSM-5 Conduct disorder			455 (84.9%)	54 (10.1%)	27 (5.0%)

* = column percentages.

### Prevalence of conduct disorder symptoms

**Figure 1:**
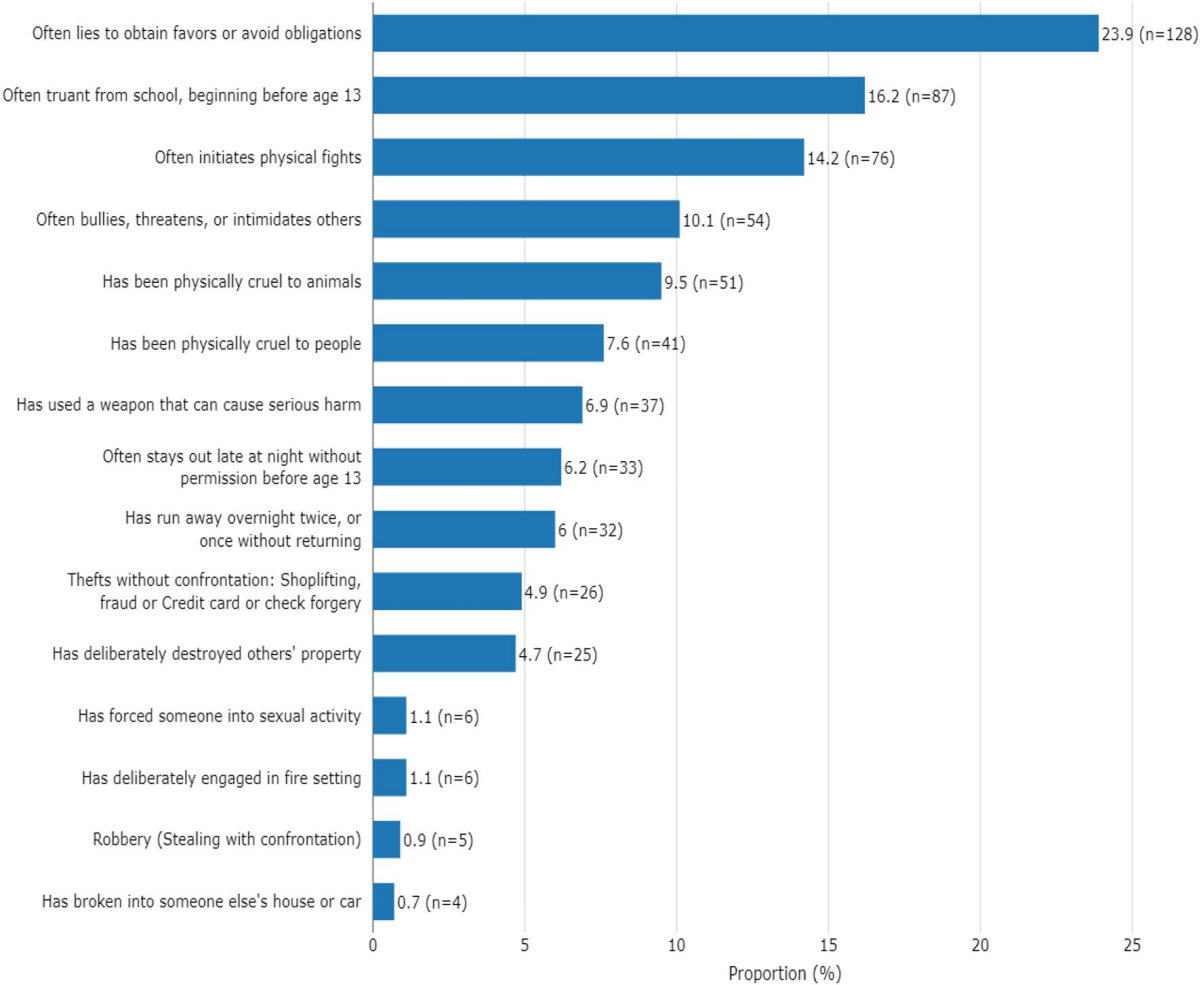
Prevalence of conduct disorder symptoms in WERCAP positive students.

Figure 1 summarizes the prevalence of CD symptoms arranged in descending order. The three leading symptoms were (i) often lie to obtain favors or avoid obligations (23.9%); (ii) often truant from school before age 13 (16.2%) and (iii) often initiate physical fights (14.2%). In addition, 10.1% often bullies, threatens or intimidates others while 9.5% have been physically cruel to animals and 7.6% of respondents have been physically cruel to people. The smallest proportion of the respondents (0.7%) has broken into someone else's house or car.

### Socio-demographics of conduct disorders

Table 2 represents the socio-demographic characteristics of the respondents. Male gender was the only characteristic significantly (*p* < 0.05) associated with CD.

**Table 2:** Social Demographics in Conduct disorder in positive WERCAP symptoms students.

Variable	Category	Total (N = 536)	Conduct disorder*			*p*-value
			Negative	Indeterminate	All criteria met except exclusion for antisocial personality	
Age (years)	15–19	136 (25.4%)	119 (87.5%)	10 (7.4%)	7 (5.1%)	0.475^‡^
	20–25	400 (74.6%)	336 (84%)	44 (11%)	20 (5%)	
Gender	Female	261 (48.7%)	231 (88.5%)	22 (8.4%)	8 (3.1%)	0.048^‡^
	Male	275 (51.3%)	224 (81.5%)	32 (11.6%)	19 (6.9%)	
Religion	Protestant	307 (57.7%)	260 (84.7%)	33 (10.7%)	14 (4.6%)	0.910^†^
	Catholic	184 (34.6%)	159 (86.4%)	16 (8.7%)	9 (4.9%)	
	Muslim	19 (3.6%)	15 (78.9%)	3 (15.8%)	1 (5.3%)	
	Other	22 (4.1%)	19 (86.4%)	2 (9.1%)	1 (4.5%)	
Birth position	1–2	297 (55.4%)	251 (84.5%)	30 (10.1%)	16 (5.4%)	0.306^†^
	3–5	190 (35.4%)	163 (85.8%)	21 (11.1%)	6 (3.2%)	
	6 +	49 (9.1%)	41 (83.7%)	3 (6.1%)	5 (10.2%)	
Level of education self	High school	61 (11.4%)	53 (86.9%)	5 (8.2%)	3 (4.9%)	0.434^†^
	College	96 (17.9%)	86 (89.6%)	5 (5.2%)	5 (5.2%)	
	University unspecified	378 (70.7%) 1(0.2%)	315 (83.3%)	44 (11.6%)	19 (5%)	
Level of education mother	Primary	106 (21.6%)	89 (84%)	10 (9.4%)	7 (6.6%)	0.840^†^
	Secondary	96 (19.6%)	80 (83.3%)	12 (12.5%)	4 (4.2%)	
	Tertiary	289 (58.9%)	247 (85.5%)	28 (9.7%)	14 (4.8%)	
Level of education father	Primary	71 (15.1%)	60 (84.5%)	7 (9.9%)	4 (5.6%)	0.851^†^
	Secondary	78 (16.6%)	69 (88.5%)	7 (9%)	2 (2.6%)	
	Tertiary	321 (68.3%)	269 (83.8%)	33 (10.3%)	19 (5.9%)	
Marital status of parents	Married	431 (80.7%)	365 (84.7%)	45 (10.4%)	21 (4.9%)	0.823^†^
	Widowed	43 (8.1%)	38 (88.4%)	2 (4.7%)	3 (7%)	
	Divorced	31 (5.8%)	26 (83.9%)	3 (9.7%)	2 (6.5%)	
	Never married	29 (5.4%)	24 (82.8%)	4 (13.8%)	1 (3.4%)	
Employment status mother	Self-employed	209 (42.0%)	183 (87.6%)	15 (7.2%)	11 (5.3%)	0.180^‡^
	Employed	124 (24.9%)	104 (83.9%)	17 (13.7%)	3 (2.4%)	
	Unemployed	165 (33.1%)	137 (83%)	17 (10.3%)	11 (6.7%)	
Employment status father	Self-employed	172 (39.4%)	156 (90.7%)	12 (7%)	4 (2.3%)	0.054^†^
	Employed	191 (43.8%)	153 (80.1%)	24 (12.6%)	14 (7.3%)	
	Unemployed	73 (16.7%)	61 (83.6%)	9 (12.3%)	3 (4.1%)	
Wealth index	Quintile 1	107 (20.0%)	90 (84.1%)	13 (12.1%)	4 (3.7%)	0.621^†^
	Quintile 2	99 (18.5%)	81 (81.8%)	9 (9.1%)	9 (9.1%)	
	Quintile 3	107 (20.0%)	95 (88.8%)	7 (6.5%)	5 (4.7%)	
	Quintile 4	107 (20.0%)	92 (86%)	11 (10.3%)	4 (3.7%)	
	Quintile 5	115 (21.5%)	96 (83.5%)	14 (12.2%)	5 (4.3%)	
Living status	Both parents	424 (82.7%)	361 (85.1%)	45 (10.6%)	18 (4.2%)	0.603^†^
	Mother only	50 (9.7%)	42 (84%)	4 (8%)	4 (8%)	
	Father only	20 (3.9%)	18 (90%)	1 (5%)	1 (5%)	
	Others	19 (3.7%)	15 (78.9%)	2 (10.5%)	2 (10.5%)	

* = row percentages; ‡ = chi-square test; † = Fisher's exact test; *p*-value = significance level.

### Conduct disorder symptoms and gender

Table 3 represents CD symptoms stratified by gender. CD symptoms were more in males than females in 13 out of 15 symptoms with 8 of the symptoms significantly (*p* < 0.05) associated with the male gender and with no gender differences in the rest of the symptoms.

**Table 3:** Gender and conduct disorder symptoms in positive WERCAP students.

Conduct disorder symptoms	Category	Total (N = 536)	Gender*		*p*-value
			Female	Male	
1. Often bullies, threatens, or intimidates others	No	482 (89.9%)	245 (93.9%)	237 (86.2%)	0.003^‡^
	Yes	54 (10.1%)	16 (6.1%)	38 (13.8%)	
2. Often initiates physical fights	No	460 (85.8%)	239 (91.6%)	221 (80.4%)	< 0.001^‡^
	Yes	76 (14.2%)	22 (8.4%)	54 (19.6%)	
3. Has used a weapon that can cause serious harm	No	499 (93.1%)	247 (94.6%)	252 (91.6%)	0.171^‡^
	Yes	37 (6.9%)	14 (5.4%)	23 (8.4%)	
4. Has been physically cruel to people	No	495 (92.4%)	248 (95.0%)	247 (89.8%)	0.024^‡^
	Yes	41 (7.6%)	13 (5.0%)	28 (10.2%)	
5. Has been physically cruel to animals	No	485 (90.5%)	245 (93.9%)	240 (87.3%)	0.009^‡^
	Yes	51 (9.5%)	16 (6.1%)	35 (12.7%)	
6. Robbery (stealing with confrontation)	No	531 (99.1%)	258 (98.9%)	273 (99.3%)	0.679^†^
	Yes	5 (0.9%)	3 (1.1%)	2 (0.7%)	
7. Has forced someone into sexual activity	No	530 (98.9%)	261 (100.0%)	269 (97.8%)	0.031^†^
	Yes	6 (1.1%)	0 (0%)	6 (2.2%)	
8. Has deliberately engaged in fire setting	No	530 (98.9%)	259 (99.2%)	271 (98.5%)	0.686^†^
	Yes	6 (1.1%)	2 (0.8%)	4 (1.5%)	
9. Has deliberately destroyed others' property	No	511 (95.3%)	254 (97.3%)	257 (93.5%)	0.034^‡^
	Yes	25 (4.7%)	7 (2.7%)	18 (6.5%)	
10. Has broken into someone else's house or car	No	531 (99.1%)	258 (98.9%)	273 (99.3%)	0.807^†^
	Yes	4 (0.8%)	2 (0.8%)	2 (0.7%)	
	Indeterminate	1 (0.2%)	1 (0.4%)	0 (0%)	
11. Often lies to obtain favors or avoid obligations	No	408 (76.1%)	204 (78.2%)	204 (74.2%)	0.280^‡^
	Yes	128 (23.9%)	57 (21.8%)	71 (25.8%)	
12. Thefts without confrontation: shoplifting, fraud or Credit card or check forgery	No	510 (95.1%)	250 (95.8%)	260 (94.5%)	0.504^‡^
	Yes	26 (4.9%)	11 (4.2%)	15 (5.5%)	
13. Often stays out late at night without permission before age 13	No	503 (93.8%)	249 (95.4%)	254 (92.4%)	0.143^‡^
	Yes	33 (6.2%)	12 (4.6%)	21 (7.6%)	
14. Has run away overnight twice, or once without returning	No	504 (94.0%)	255 (97.7%)	249 (90.5%)	< 0.001^‡^
	Yes	32 (6.0%)	6 (2.3%)	26 (9.5%)	
15. Often truant from school, beginning before age 13	No	449 (83.8%)	231 (88.5%)	218 (79.3%)	0.004^‡^
	Yes	87 (16.2%)	30 (11.5%)	57 (20.7%)	

* = column percentages; ‡ = chi-square test; † = Fisher's exact test; *p*-value = significance level.

### Conduct disorder and mental disorders

Table 4 represents the mental health disorders of the respondents. Obsessive compulsive disorder, psychosis, agoraphobia, social phobia, drug abuse/dependence, suicidality, WERCAP bipolar disorder and WERCAP schizophrenia were significantly (*p* < 0.05) associated with CD. Those with DSM-5 syndromes of alcohol and substance use/dependence respectively had comorbid diagnosis of CD in 5.4% and 6.2% respectively.

**Table 4:** DSM 5 Mental disorders and DSM 5 Conduct disorder in WERCAP positive students.

Variable	Category	Total (N = 536)	Conduct disorder*			*p*-value
			Negative	Indeterminate	All criteria met	
Major depressive disorder	No	358 (66.8%)	312 (87.2%)	31 (8.7%)	15 (4.2%)	0.115^‡^
	Yes	178 (33.2%)	143 (80.3%)	23 (12.9%)	12 (6.7%)	
PTSD	No	336 (62.7%)	290 (86.3%)	31 (9.2%)	15 (4.5%)	0.486^‡^
	Yes	200 (37.3%)	165 (82.5%)	23 (11.5%)	12 (6%)	
Bulimia/binge eating disorder	No	495 (92.4%)	423 (85.5%)	50 (10.1%)	22 (4.4%)	0.090^†^
	Yes	41 (7.6%)	32 (78%)	4 (9.8%)	5 (12.2%)	
Obsessive compulsive disorder	No	175 (32.6%)	157 (89.7%)	15 (8.6%)	3 (1.7%)	0.030^‡^
	Yes	361 (67.4%)	298 (82.5%)	39 (10.8%)	24 (6.6%)	
Panic disorder	No	378 (70.5%)	327 (86.5%)	36 (9.5%)	15 (4%)	0.158^‡^
	Yes	158 (29.5%)	128 (81%)	18 (11.4%)	12 (7.6%)	
Psychosis	No	262 (48.9%)	239 (91.2%)	17 (6.5%)	6 (2.3%)	< 0.001^‡^
	Yes	274 (51.1%)	216 (78.8%)	37 (13.5%)	21 (7.7%)	
Agoraphobia	No	305 (57.0%)	271 (88.9%)	24 (7.9%)	10 (3.3%)	0.015^‡^
	Yes	230 (43.0%)	184 (80%)	30 (13%)	16 (7%)	
Social phobia	No	229 (42.7%)	201 (87.8%)	23 (10%)	5 (2.2%)	0.032^‡^
	Yes	307 (57.3%)	254 (82.7%)	31 (10.1%)	22 (7.2%)	
Alcohol abuse/dependence	No	388 (72.4%)	336 (86.6%)	33 (8.5%)	19 (4.9%)	0.137^‡^
	Yes	148 (27.6%)	119 (80.4%)	21 (14.2%)	8 (5.4%)	
Drug abuse/dependence	No	407 (75.9%)	354 (87%)	34 (8.4%)	19 (4.7%)	0.043^‡^
	Yes	129 (24.1%)	101 (78.3%)	20 (15.5%)	8 (6.2%)	
Generalized anxiety disorder	No	419 (78.2%)	360 (85.9%)	39 (9.3%)	20 (4.8%)	0.444^‡^
	Yes	117 (21.8%)	95 (81.2%)	15 (12.8%)	7 (6%)	
Somatization disorder	No	364 (67.9%)	312 (85.7%)	36 (9.9%)	16 (4.4%)	0.590^‡^
	Yes	172 (32.1%)	143 (83.1%)	18 (10.5%)	11 (6.4%)	
Hypochondriasis	No	340 (63.4%)	292 (85.9%)	33 (9.7%)	15 (4.4%)	0.620^‡^
	Yes	196 (36.6%)	163 (83.2%)	21 (10.7%)	12 (6.1%)	
Suicidality	No	498 (92.9%)	428 (85.9%)	46 (9.2%)	24 (4.8%)	0.030^†^
	Yes	38 (7.1%)	27 (71.1%)	8 (21.1%)	3 (7.9%)	
Positive screen (not diagnosis) for high risk WERCAP Bipolar Disorder	Mean (SD)	14.84 (11.53)	14.31 (11.43)	17.26 (12.01)	18.93 (11.24)	0.032^a^
WERCAP Schizophrenia	Mean (SD)	18.70 (18.21)	17.79 (17.98)	23.46 (19.25)	24.63 (18.05)	0.028^a^

* = row percentages; a = Kruskal–Wallis test; ‡ = chi-square test; † = Fisher's exact test; *p*-value = significance level.

### Conduct disorder and substance use

Table 5 represents the ASSIST lifetime and current substance use (not necessarily dependency) of the respondents. All drugs except hallucinogens lifetime were significantly (*p* < 0.05) associated with CD.

**Table 5:** ASSIST Substance use (Lifetime and Current) and DSM 5 conduct disorders.

Variable	Category	Total (N = 536)	Conduct disorder*			*p*-value
			Negative	Indeterminate	All criteria met except	
Tobacco lifetime	No	480 (89.6%)	417 (86.9%)	42 (8.8%)	21 (4.4%)	0.001^†^
	Yes	56 (10.4%)	38 (67.9%)	12 (21.4%)	6 (10.7%)	
Alcohol lifetime	No	406 (75.7%)	354 (87.2%)	35 (8.6%)	17 (4.2%)	0.031^‡^
	Yes	130 (24.3%)	101 (77.7%)	19 (14.6%)	10 (7.7%)	
Cannabis lifetime	No	491 (92.1%)	423 (86.2%)	47 (9.6%)	21 (4.3%)	0.006^†^
	Yes	42 (7.9%)	29 (69%)	7 (16.7%)	6 (14.3%)	
Cocaine lifetime	No	524 (98.1%)	448 (85.5%)	51 (9.7%)	25 (4.8%)	0.010^†^
	Yes	10 (1.9%)	5 (50%)	3 (30%)	2 (20%)	
Amphetamine lifetime	No	523 (97.8%)	448 (85.7%)	51 (9.8%)	24 (4.6%)	0.004^†^
	Yes	12 (2.2%)	6 (50%)	3 (25%)	3 (25%)	
Inhalants lifetime	No	528 (98.7%)	450 (85.2%)	53 (10%)	25 (4.7%)	0.028^†^
	Yes	7 (1.3%)	4 (57.1%)	1 (14.3%)	2 (28.6%)	
Sedatives lifetime	No	513 (95.9%)	443 (86.4%)	46 (9%)	24 (4.7%)	< 0.001^†^
	Yes	22 (4.1%)	11 (50%)	8 (36.4%)	3 (13.6%)	
Hallucinogens lifetime	No	526 (98.3%)	448 (85.2%)	52 (9.9%)	26 (4.9%)	0.171^†^
	Yes	9 (1.7%)	6 (66.7%)	2 (22.2%)	1 (11.1%)	
Opioids lifetime	No	524 (97.9%)	448 (85.5%)	50 (9.5%)	26 (5%)	0.012^†^
	Yes	11 (2.1%)	6 (54.5%)	4 (36.4%)	1 (9.1%)	
Khat lifetime	No	519 (96.8%)	446 (85.9%)	51 (9.8%)	22 (4.2%)	< 0.001^†^
	Yes	17 (3.2%)	9 (52.9%)	3 (17.6%)	5 (29.4%)	
Tobacco current	No	502 (93.7%)	435 (86.7%)	46 (9.2%)	21 (4.2%)	< 0.001^†^
	Yes	34 (6.3%)	20 (58.8%)	8 (23.5%)	6 (17.6%)	
Alcohol current	No	429 (80.0%)	374 (87.2%)	38 (8.9%)	17 (4%)	0.009^‡^
	Yes	107 (20.0%)	81 (75.7%)	16 (15%)	10 (9.3%)	
Cannabis current	No	504 (94.0%)	434 (86.1%)	49 (9.7%)	21 (4.2%)	0.002^†^
	Yes	32 (6.0%)	21 (65.6%)	5 (15.6%)	6 (18.8%)	
Cocaine current	No	531 (99.1%)	453 (85.3%)	53 (10%)	25 (4.7%)	0.008^†^
	Yes	5 (0.9%)	2 (40%)	1 (20%)	2 (40%)	
Amphetamine current	No	526 (98.1%)	450 (85.6%)	51 (9.7%)	25 (4.8%)	0.010^†^
	Yes	10 (1.9%)	5 (50%)	3 (30%)	2 (20%)	
Inhalants current	No	533 (99.4%)	455 (85.4%)	53 (9.9%)	25 (4.7%)	< 0.001^†^
	Yes	3 (0.6%)	0 (0%)	1 (33.3%)	2 (66.7%)	
Sedatives current	No	523 (97.6%)	451 (86.2%)	48 (9.2%)	24 (4.6%)	< 0.001^†^
	Yes	13 (2.4%)	4 (30.8%)	6 (46.2%)	3 (23.1%)	
Hallucinogens current	No	533 (99.4%)	454 (85.2%)	52 (9.8%)	27 (5.1%)	0.035^†^
	Yes	3 (0.6%)	1 (33.3%)	2 (66.7%)	0 (0%)	
Opioids current	No	530 (98.9%)	453 (85.5%)	51 (9.6%)	26 (4.9%)	0.007^†^
	Yes	6 (1.1%)	2 (33.3%)	3 (50%)	1 (16.7%)	
Khat current	No	526 (98.1%)	450 (85.6%)	52 (9.9%)	24 (4.6%)	0.004^†^
	Yes	10 (1.9%)	5 (50%)	2 (20%)	3 (30%)	

* = row percentages; ‡ = chi-square test; † = Fisher's exact test; *p*-value = significance level.

### Independent predictors of conduct disorder

Table 6 represents the independent predictors of CD.

**Table 6:** Independent Predictors of Conduct disorder.

Variable	Category	Model 1	Model 2
		AOR (95% CI)	AOR (95% CI)
Gender	Female	Ref	Ref
	Male	1.48 (1.24–1.77)***	1.54 (1.29–1.84)***
Obsessive compulsive disorder	No	Ref	Ref
	Yes	1.17 (0.93–1.48)	1.17 (0.93–1.47)
Psychosis	No	Ref	Ref
	Yes	1.29 (1.03–1.63)*	1.25 (0.99–1.59)
Agoraphobia	No	Ref	Ref
	Yes	0.94 (0.75–1.18)	0.94 (0.75–1.18)
Social phobia	No	Ref	Ref
	Yes	0.99 (0.79–1.24)	1.04 (0.83–1.31)
Drug abuse/dependence	No	Ref	Ref
	Yes	1.06 (0.87–1.30)	1.06 (0.87–1.29)
Suicidality	No	Ref	Ref
	Yes	0.75 (0.55–1.00)	0.78 (0.57–1.04)
Tobacco lifetime	No	Ref	–
	Yes	1.66 (1.20–2.28)**	–
Alcohol lifetime	No	Ref	–
	Yes	0.95 (0.74–1.22)	–
Cannabis lifetime	No	Ref	–
	Yes	0.65 (0.47–0.89)**	–
Cocaine lifetime	No	Ref	–
	Yes	0.98 (0.51–1.81)	–
Amphetamine lifetime	No	Ref	–
	Yes	1.00 (0.52–1.89)	–
Inhalants lifetime	No	Ref	–
	Yes	1.34 (0.62–2.83)	–
Sedatives lifetime	No	Ref	–
	Yes	0.86 (0.50–1.43)	–
Opioids lifetime	No	Ref	–
	Yes	2.48 (1.43–4.08)***	–
Khat lifetime	No	Ref	–
	Yes	1.00 (0.66–1.50)	–
Tobacco current	No	–	Ref
	Yes	–	1.30 (0.92–1.84)
Alcohol current	No	–	Ref
	Yes	–	1.03 (0.80–1.31)
Cannabis current	No	–	Ref
	Yes	–	0.68 (0.48–0.96)*
Cocaine current	No	–	Ref
	Yes	–	1.27 (0.54–2.75)
Amphetamine current	No	–	Ref
	Yes	–	0.88 (0.38–1.80)
Inhalants current	No	–	Ref
	Yes	–	1.89 (0.74–5.00)
Sedatives current	No	–	Ref
	Yes	–	1.01 (0.58–1.71)
Hallucinogens current	No	–	Ref
	Yes	–	0.61 (0.21–1.64)
Opioids current	No	–	Ref
	Yes	–	3.08 (1.61–5.49)***
Khat current	No	–	Ref
	Yes	–	0.88 (0.55–1.39)
WERCAP bipolar disorder	Mean (SD)	1.01 (0.99–1.02)	1.01 (0.99–1.02)
WERCAP Schizophrenia	Mean (SD)	1.00 (0.99–1.01)	1.00 (0.99–1.01)

AOR = Adjusted Odds Ratio; CI = confidence interval; *p < 0.05; **p < 0.01; ***p < 0.001; model 1 = Poisson regression where current substance use for Tobacco, alcohol, cannabis, cocaine, amphetamine, inhalants, sedatives, Hallucinogens, opioids and Khat are omitted; model 2 = Poisson regression where lifetime substance use for tobacco, alcohol, cannabis, cocaine, amphetamine, inhalants, sedatives, opioids and khat are omitted.

In model 1, being male, psychosis, tobacco lifetime use, cannabis lifetime use and opioids lifetime use were independent predictors (*p* < 0.05) of CD.

In model 2, being male, cannabis current use and opioids current use were independent predictors (*p* < 0.05) of CD.

## Discussion

Here we present the first study that examines a wide range of DSM-5 CD symptoms and diagnosis in a cohort of Kenyan students who had screened positive for psychosis, affectivity and had various mental disorders and substance use disorders. This was not an epidemiological study of CD symptoms and disorder. Rather it was a study that sought to highlight the potential comorbidity of CD symptoms and disorders in persons with DSM-5 diagnosis of mental disorders and substance use and the WHO ASSIST substance use. Unlike DSM-5, WHO ASSIST gives a wide range of substance use disorders. We make the distinction between substance use/dependence disorders as defined by DSM-5 and substance use – as defined by ASSIST.

We find that CD is a potential differential disorder in youth with mental and substance use disorders and psychosis. This comorbidity may complicate the outcome of each of these disorders if not identified and managed.

Our findings concur with findings from other countries including USA reviewed under the introduction, that CD and substance use are co-morbid. We studied high risk psychotic participants, some of them expected to convert to DSM-5 psychotic disorders. The findings are significant in youth when most mental disorders begin. Additionally, the finding of high risk participants not only points to the possibility of schizophrenia but also CD and substance use, where early intervention is necessary. The focus is on the youth with the aim to give them a comprehensive and inclusive management.

Our findings of definite and indeterminate CD are similar to what was reported in a 2020 epidemiological study in Nigeria in patients without a history of psychosis reviewed under the introduction. Unlike the Nigeria study that was on inpatients, our study was on community-based students. Either way in respect to prevalence, whether clinical patients or community participants, the definite DSM-5 diagnosis clearly illustrates the magnitude and the need to identify and manage CD at the earliest possible time more so given that the 2^nd^ commonest symptoms started before the age of 13.

In agreement with global Africa and Kenyan literature cited in the introduction, our findings demonstrate that all DSM-5 CD and its various symptoms occur in both genders but are significantly associated with the male gender in the majority. It is noteworthy that in our study, apart from gender none of the symptoms of CD were significantly associated with all the other socio-demographic variables. This is in contrast with the finding reviewed in the literature that CD was more associated with early age. We have no explanation for this finding in our study. In addition, of note is that even the socioeconomic status of the family as measured by the wealth index was not associated with CD.

Our study and findings do not allow us to make any inference about the cause effect relationships between the various variables. Nevertheless, they have both policy and practice implications. They strongly suggest that in any attempts to screen for psychosis in youth, attempts should be made to also screen for CD, mental disorders and substance use and manage them in an integrated approach. There is a possibility that the expression of CD could be influenced by culture. However, in our case we used DSM 5 criteria which are universal and for the purposes of comparison with other studies. It is noteworthy that CD, despite the positive screen for psychosis, was associated with nearly all substance use disorders. However, this association is widely documented even in none psychotic disorders. We speculate that the finding that cannabis was least associated with CD is a reflection of the increased acceptability of cannabis use in youth. There is increasing pressure to liberate the use of cannabis.

### Strength and limitation

A major limitation of this study is the cross-sectional nature of the study thus no cause-effect relationships could be established. All the participants were from institutions of learning and not community based populations thus a need for community based youth to be studied to complete the picture. The strength of this study is the representation of various levels of the youth, and the multiple variables in the same cohort.

## Conclusion

There is a need for Kenyan prospective study or studies in community non-institutional settings. There is also a need for clinical population studies.

## Data Availability

The datasets used and/or analyzed during the current study are available from the corresponding author upon reasonable request.
